# Double-Layer Compressive Sensing Based Efficient DOA Estimation in WSAN with Block Data Loss

**DOI:** 10.3390/s17071688

**Published:** 2017-07-22

**Authors:** Peng Sun, Liantao Wu, Kai Yu, Huajie Shao, Zhi Wang

**Affiliations:** 1 State Key Laboratory of Industrial Control Technology, Zhejiang University, Hangzhou 310027, China; sunpengzju@zju.edu.cn (P.S.); wuliantao@zju.edu.cn (L.W.); kaiyuzju@gmail.com (K.Y.); 2 Department of Computer Science, University of Illinois at Urbana-Champaign, Urbana, IL 61801, USA; hshao5@illinois.edu

**Keywords:** double-layer compressive sensing, direction of arrival, block data loss, packet size, joint sparse representation

## Abstract

Accurate information acquisition is of vital importance for wireless sensor array network (WSAN) direction of arrival (DOA) estimation. However, due to the lossy nature of low-power wireless links, data loss, especially block data loss induced by adopting a large packet size, has a catastrophic effect on DOA estimation performance in WSAN. In this paper, we propose a double-layer compressive sensing (CS) framework to eliminate the hazards of block data loss, to achieve high accuracy and efficient DOA estimation. In addition to modeling the random packet loss during transmission as a passive CS process, an active CS procedure is introduced at each array sensor to further enhance the robustness of transmission. Furthermore, to avoid the error propagation from signal recovery to DOA estimation in conventional methods, we propose a direct DOA estimation technique under the double-layer CS framework. Leveraging a joint frequency and spatial domain sparse representation of the sensor array data, the fusion center (FC) can directly obtain the DOA estimation results according to the received data packets, skipping the phase of signal recovery. Extensive simulations demonstrate that the double-layer CS framework can eliminate the adverse effects induced by block data loss and yield a superior DOA estimation performance in WSAN.

## 1. Introduction

As a branch of array signal processing, DOA estimation has been a hot topic in many research fields, such as smart antennas, mobile communication and target tracking [1,2,3,4,5]. Traditionally, DOA estimation is implemented using sensor arrays, such as in active sonar systems [6,7], where all sensors have a wired connection to the fusion center (FC). For wireless sensor array networks (WSAN) [8,9,10], sensor arrays are deployed in a large sensor field and communicate with the FC via wireless channels. Generally, the data transmission process from sensor arrays to the FC is assumed to be reliable and lossless.

Unfortunately, due to the existence of channel noise, multi-path effects and link asymmetry, etc., low-power wireless links often generate a high packet loss rate [11]. Data sent from the sensor array to the FC are often subjected to missing or garbled values, posing a great challenge for information acquisition at the FC. Thanks to the sparsity of monitored signals, the newly-emerged compressive sensing (CS) technique has a broad application prospect in wireless communication because of its ability to recover the raw signal from a small number of random measurements [12,13,14,15]. Additionally, it has been widely used to deal with data loss in wireless sensor networks (WSNs). In [16], the authors presented the low-rank structure, spatial similarity and temporal stability of the environmental data and proposed a novel approach based on CS to reconstruct the lost data. In [17], a CS-based lost data recovery approach for smart wireless accelerometers used in structural health monitoring (SHM) was proposed, and the raw acceleration signal can be effectively reconstructed, though some data loss may happen. In [18], an oversampled CS source coding was adopted to neutralize the stochastic nature of wireless link disturbances and hence compensate channel erasures, making the data recovery at the sink largely immune to data loss. The work in [19] studied the lossy nature of low-power wireless links and introduced a CS-based channel coding scheme. Utilizing CS reconstruction techniques rather than traditional interpolation methods for lost data recovery, they achieved an enhanced channel utilization and transmission reliability. However, all of these works demonstrated their results in terms of the accuracy of signal recovery, without insight into the level of information acquisition, e.g., the DOA information in WSAN. Besides, there is an issue that has not been investigated yet. Block data loss is common in wireless communication, as data samples are transmitted in the form of packets, and a large packet size is often adopted for high transmission efficiency. However, block data loss often does great harm to various applications.

In this paper, we first investigate the adverse effects of block data loss on DOA estimation in WSAN. Subsequently, a double-layer compressive sensing framework is proposed to eliminate its hazards and to realize high accuracy and efficient DOA estimation. Under the double-layer CS framework, DOA estimation is implemented according to the received data samples (the measurement vectors) at the FC. Conventionally, DOA estimation is fulfilled in two stages. We first need to recover the raw sparse vectors and then perform DOA estimation based on them. This process is denoted by DCS-DOA. Furthermore, to avoid the error propagation from signal recovery to DOA estimation in DCS-DOA, we propose a direct DOA estimation technique, called DCS-DDOA, to achieve a better DOA estimation performance. Leveraging a joint frequency and spatial domain sparse representation of the sensor array data, the FC can directly obtain the DOA estimation results according to the same received data samples, skipping the stage of signal recovery. Extensive simulations are carried out to validate that the double-layer CS framework can dispel the detriment of block data loss and yield a superior DOA estimation performance in WSAN.

The main contributions of this paper are summarized as follows:

- A double-layer compressive sensing framework is proposed to eliminate the adverse effects of block data loss on DOA estimation in WSAN. Specifically, we model the random packet loss during transmission as a passive CS process and introduce an active CS process at each array sensor to address the block data loss problem.
- We present the mutual coherence of the equivalent measurement matrix and the absolute off-diagonal entries’ distribution of the corresponding Gram matrix under the double-layer CS framework, which account for the satisfactory DOA estimation performance.
- A direct DOA estimation technique (DCS-DDOA) is proposed under the double-layer CS framework to avoid the error propagation problem in DCS-DOA. A joint frequency and spatial domain sparse representation of the sensor array data is constructed and exploited to directly perform DOA estimation at the FC.

The remainder of this paper is organized as follows. Section 2 describes the preliminaries of compressive sensing, array signal model, lossy wireless links and block data loss briefly. In Section 3, we elaborate the double-layer CS framework and the two DOA estimation techniques. The performance evaluation for DCS-DOA and DCS-DDOA is presented in Section 4, followed by conclusions in Section 5.

## 2. Background

### 2.1. Compressive Sensing

Denote x=Ψs, where $x\in R^{N\times 1}$ is the signal of interest, and $\Psi \in R^{N\times N}$ is a transformation basis. We say x is *K*-sparse if s has only $K\left(K\ll N\right)$ dominant elements, while other elements are zero or close to zero. The theory of CS states that, under certain conditions, instead of periodically sampling x, we only need to acquire $M\left(M\ll N\right)$ non-adaptive linear measurements y=Φx, where $\Phi \in R^{M\times N}$ is a carefully-chosen measurement matrix [15]. Combining the measurement process and sparse representation, we have y=Φx=ΦΨs=As, where A is called the equivalent sensing matrix. It was shown in [20] that x can be exactly recovered from its measurement vector y by solving the following constrained optimization problem:

(1) $$\min\;\;\;{∥s∥}_{0}\;\;\;\;\;\;\;\;\;\;\;\;\;s.t.\;\;\;\;\;\;\;\;\;\;y=\mathrm{As}$$

as long as $K<\frac{1}{2}\left(1+\frac{1}{\mu \left(A\right)}\right)$. Additionally, $\mu \left(A\right)=\max_{i\neq j,1\leq i,j\leq N}\left\{\;\;\frac{\left|a_{i}^{T}a_{j}\right|}{∥a_{i}∥\cdot ∥a_{j}∥}\;\;\right\}$ represents the mutual coherence of A, with $a_{i}$, $a_{j}$ denoting the *i*-th and *j*-th column, respectively. Another metric is the average mutual coherence $\mu_{av}\left(A\right)=\underset{i\neq j,1\leq i,j\leq N}{\mathrm{mean}}\left\{\frac{\left|{a_{i}}^{T}a_{j}\right|}{∥a_{i}∥\cdot ∥a_{j}∥}\right\}$. They can also be determined according to the maximum or average value among the absolute off-diagonal elements in the corresponding Gram matrix $G={\tilde{A}}^{T}\tilde{A}$, where $\tilde{A}$ is the column-normalized version of A. The mutual coherence $\mu \left(A\right)$ is by now a classical way of analyzing the recovery abilities of a measurement matrix. Furthermore, it can be proven that the solution to the problem in (1) is the same as the relaxed one to the $l_{1}$-based minimization below:

(2) $$\min\;\;\;{∥s∥}_{1}\;\;\;\;\;\;\;\;\;\;\;\;\;s.t.\;\;\;\;\;\;\;\;\;\;y=\mathrm{As}$$

which can be solved efficiently using the algorithms such as basis pursuit (BP) [15].

### 2.2. DOA Estimation

Without loss of generality, we consider a uniform linear array (ULA) consisting of *H* omnidirectional sensors, with an inter-element spacing of *d*, d<λminλmin22, and $\lambda_{\min}$ is the minimum wavelength of the impinging signals. Consider *K* far-field wideband signals $s_{k}\left(t\right)$ incident on the ULA from direction $\theta_{k}$, k=1,…,K. The array output vector $x\left(t\right)$ is then given by:

(3) $$x\left(t\right)=\sum_{k=1}^{K}a\left(\theta_{k}\right)s_{k}\left(t\right)+n\left(t\right),\;\;\;\;\;\;\;t=1,\ldots ,T$$

where $a\left(\theta_{k}\right)={\left[1,e^{-j2\pi f\frac{d\sin\left(\theta_{k}\right)}{c}},\ldots ,e^{-j2\pi f\frac{\left(H-1\right)d\sin\left(\theta_{k}\right)}{c}}\right]}^{T}$ is the H×1 steering vector of the array corresponding to $\theta_{k}$; $A=\left[a\left(\theta_{1}\right),\ldots ,a\left(\theta_{K}\right)\right]$ is the H×K array manifold matrix; $s\left(t\right)={\left[s_{1}\left(t\right),\ldots ,s_{K}\left(t\right)\right]}^{T}$ is the source signal vector. *T* is the number of samples; *c* is the wave speed. $n\left(t\right)$ is the noise vector, whose elements are assumed to be temporally and spatially white, and uncorrelated from the sources.

Observing that signals impinging on the arrays are intrinsically sparse in the spatial domain, efficient strategies for DOA estimation based on sparse signal recovery have been proposed in [3,21], where the sparsity constraints have been enforced through a $l_{1}$-norm minimization. The concept of group-sparsity has been exploited to cope with the problems of wideband DOA estimation [22,23].

### 2.3. Lossy Wireless Links

**Figure 1:**
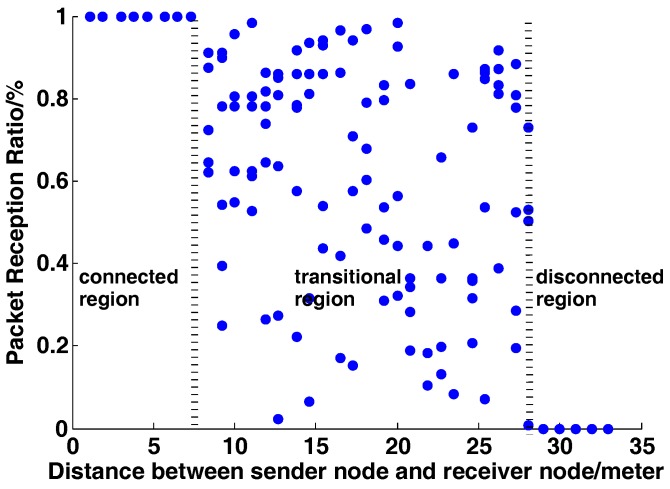
The spatial characteristic of wireless links.

Due to factors like channel noise, multi-path effects, link asymmetry, etc., low-power wireless links often suffer from a high packet loss rate [11]. We conduct extensive experiments to test the spatial characteristic of wireless links using a pair of STM32W108 chips in our teaching building corridor. Results demonstrate that wireless links can be represented by three different communication regions, the connected region, the transitional region and the disconnected region [24], as shown in Figure 1, which depicts the variation trend of packet reception rate versus the communication distance. In most cases, wireless data transmission occurs in the transitional region. Therefore, to ensure the reliability of communication, we need to take some error correcting measures, such as Automatic Repeat reQuest (ARQ), which retransmits the lost data packets, and Forward Error Correction (FEC), which adopts some coding schemes to improve the robustness of transmission. However, both of the methods above are imperfect because of the additional energy consumption and transmission delay.

Thanks to the inherent sparsity of monitored signals, compressive sensing has been widely used in WSNs. Many research works have utilized it to deal with data loss in wireless communication to improve transmission reliability. However, the emphasis was put on the accuracy of signal recovery, rather than the information acquisition in specific application scenarios.

### 2.4. Block Data Loss

**Figure 2:**
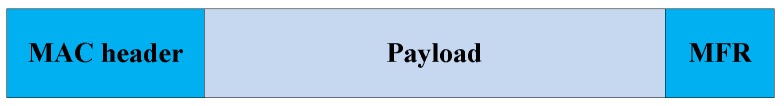
Data packet structure of IEEE 802.15.4. MFR, MAC footer.

**Figure 3:**
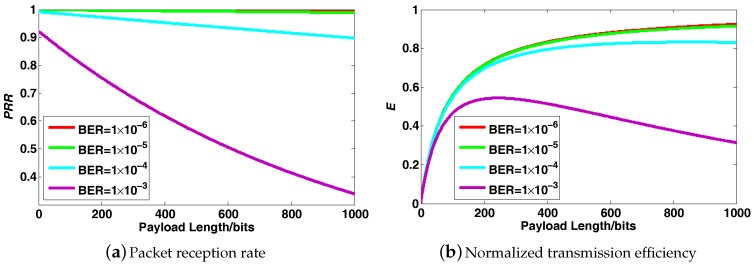
Packet reception rate (*PRR*) and normalized transmission efficiency *E* versus payload length under different *BER*.

Taking the IEEE 802.15.4 standard as an example, its data packet structure is shown in Figure 2, which consists of the MAC header (MHR), the MAC footer (MFR) and payload. Each transmitted packet has a minimum fixed overhead provided by MHR and MFR. This cost is predetermined and independent of the packet payload size. Intuitively, it is easy to come to the conclusion that using larger packet sizes could achieve a better data transmission efficiency by minimizing the overhead per useful bit transmitted in the payload. However, things may change greatly in practice. Given a bit error rate BER, the packet reception rate (PRR) can be obtained as follows:

(4) $$PRR={\left(1-BER\right)}^{L}$$

where $L=L_{payload}+L_{overhead}$ is the whole packet size including two parts: $L_{payload}$ is the payload size, and $L_{overhead}$ denotes the size of fixed overhead. Naturally, the relationship between PRR and payload size under a varying BER is illustrated in Figure 3a, achieved through simulation. Obviously, PRR falls as payload size rises, and the downtrend becomes sharper when BER increases. On the other hand, according to [25], we can define the data transmission efficiency as follows:

(5) $$E=\frac{L_{payload}\times {\left(1-BER\right)}^{L_{payload}+L_{overhead}}}{L_{payload}+L_{overhead}}$$

where *E* represents the normalized data transmission efficiency. Based on Equation (5), the relationship between *E* and $L_{payload}$ under a varied BER is depicted in Figure 3b.

**Figure 4:**
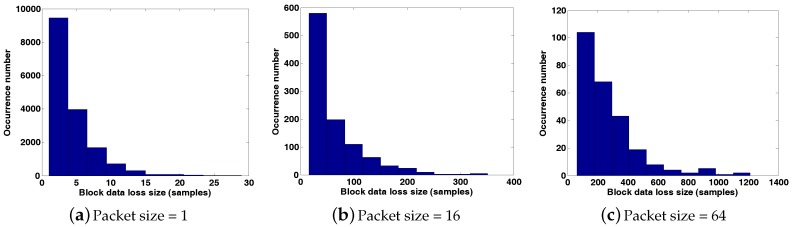
Histograms of the occurrence number for different sizes of block data loss under different packet sizes.

We will take it for granted that the data transmission efficiency will improve with a larger payload size, and this is indeed the case when BER is sufficiently low, that is wireless links are of high quality. However, in practice, BER is usually somewhat high; thus, the data transmission efficiency will first rise and then drop with the increase of payload size. This is because data packets with larger sizes are more likely to be lost, exactly as demonstrated in Figure 3a. When a packet with a large size is lost during transmission, all of the data samples in it are missing simultaneously, which is called block data loss. Block data loss does serious harm to the transmission performance, and it becomes more grievous with the increased packet size. For example, assume 65,536 data samples are transmitted over a lossy link, with PRR being 25%; the histograms of the occurrence number for different sizes of block data loss using different packet sizes are shown in Figure 4. Obviously, the size of block data loss becomes larger when a larger packet size is adopted. Therefore, there is a tradeoff between the desire to improve transmission efficiency by using a large packet size and the need to heighten the PRR and avoid block data loss by using a small packet size. Briefly, it is a meaningful thing to find some new error-correcting techniques to eliminate the hazards of block data loss, such that the data transmission efficiency is optimized.

## 3. Double-Layer CS Framework-Based DOA Estimation

**Figure 5:**
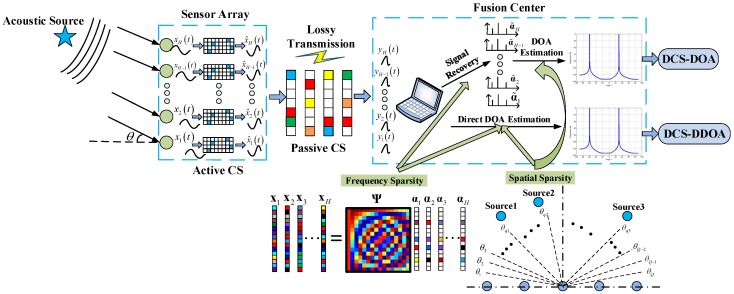
Schematic diagram of the double-layer compressive sensing (CS) framework and the DOA estimation process of DCS-DOA and DCS-direct DOA (DDOA).

In this paper, we focus on how to obtain high accuracy and efficient DOA estimation in a WSAN, which consists of an *H*-element ULA and one single FC. The array sensors are connected to the FC via low-power wireless links. Array sensors are battery powered and have limited computational capacities, while the FC is linked to the infrastructure and able to bear heavy processing tasks. Therefore, it is essential to shift as much of the processing burden to the FC as possible. Specifically, the array sensors just sample the impinging signals, perform computationally-simple operations when needed and transmit the data to the FC, while the complicated DOA estimation algorithm is implemented at the FC. Due to the lossy nature of low-power wireless links, the transmission process from the sensor array to the FC is subjected to a high packet loss rate. Data loss, especially block data loss resulting from a data packet with a large size being lost, damnifies the WSAN DOA estimation performance significantly. To eliminate the adverse effects of block data loss on DOA estimation, we propose the double-layer CS framework. The random packet loss during transmission is modeled as a passive CS process, and an active CS process is introduced at each array sensor to do a dimension-reduced projection on the acquired raw signal before transmission. Under the double-layer CS framework, DOA estimation can be implemented using two techniques, named DCS-DOA and DCS-DDOA, respectively. The entire process is described in Figure 5. For clarity, the notations are summarized in Table 1.

### 3.1. Double-Layer CS Framework

In this part, we elaborate the double-layer CS framework, which consists of a passive CS process modeling the random packet loss and an active CS process introduced at each array sensor. Furthermore, we present the mutual coherence of the equivalent measurement matrix and the absolute off-diagonal entries’ distribution of the corresponding Gram matrix, which account for the satisfactory DOA estimation performance under the double-layer CS framework.

**Figure 6:**
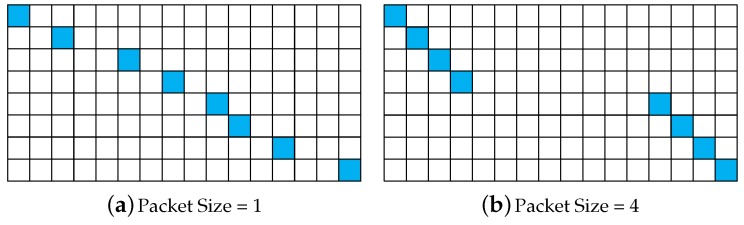
Examples of the passive CS measurement matrix under different packet sizes, with blue grids denoting one and blank grids denoting zero.

**Figure 7:**
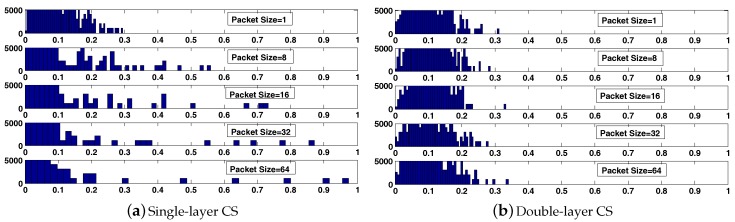
Histograms of the absolute off-diagonal elements of the corresponding Gram matrix to the equivalent measurement matrices under the single-layer CS and double-layer CS framework when *N* = 512 and *M* = 64.

For comparison, we first present a single-layer CS framework. Assume *N* data samples are acquired at each array sensor, and they are transmitted to the FC to complete one DOA estimate. Under the single-layer CS framework, the raw data samples are directly assembled into packets for transmission. The lossy nature of low-power wireless links is exploited by modeling the random packet loss during transmission as a passive CS process. Additionally, the passive CS measurement matrix, denoted by $\Phi_{r}\in R^{M\times N}$ (*M* is the number of received data samples from each array sensor), is constructed as follows:

(6) $$\Phi_{r}\left(i,j\right)=\left\{\begin{array}{c} 1\;\;\;\;\;\;\;\;\mathrm{if}\;\;\;\;\;\;j=J\left(i\right)\leq N\\ 0\;\;\;\;\;\;\;\;\;\;\;\;\;\;\;\;\;\;\;\;\mathrm{otherwise} \end{array}\right.$$

where *i* is the row index and also the sequence number of the received data samples, *j* is the column index and $J\left(i\right)$ is the corresponding sequence number in the transmitted data vector of received data. The passive CS measurement matrix is determined by the packet reception rate and the packet size (in this paper, we ignore the overhead produced by MHR and MFR and refer to the number of data samples in each packet as the packet size). For example, assume that there are 16 data samples to be transmitted over a lossy link, with the packet reception rate being 50%. When the packet size is one (only one data sample in each packet), the 16 data samples will be assembled into 16 packets for transmission, and eight packets are received with the corresponding packet number being 1, 3, 6, 8, 10, 11, 13, 16; that is, $J=\left\{1,3,6,8,10,11,13,16\right\}$. Then, the passive CS measurement matrix can be depicted in Figure 6a. When the packet size is increased to four (four data samples in each packet) for a higher transmission efficiency, the 16 data samples will be assembled into four packets for transmission. Under the same packet reception rate, two packets are received with the corresponding packet number being one and four. In this case, eight data samples are received, but $J=\left\{1,2,3,4,13,14,15,16\right\}$. Thus, the passive CS measurement matrix is depicted in Figure 6b. Obviously, when the packet size is larger than one, block data loss will emerge when a packet is missing during transmission. Additionally, this is specifically exhibited in the distribution of the element “1” of the passive CS measurement matrix. The “1”s will be less dispersive when the packet size is larger. Note that the equivalent measurement matrix is $A=\Phi_{r}\Psi$ under the single-layer CS framework. To account for the hazards of block data loss, we present the histograms of the absolute off-diagonal elements of the corresponding Gram matrix under different packet sizes. As shown in Figure 7a, when the packet size is increased from 1–64, the number of very correlated columns of the equivalent measurement matrix A is increased greatly, so the distribution of entries keeps moving towards the larger side. As a consequence, the DOA estimation performance gradually deteriorates with an increased packet size, which will be discussed in our simulations later.

**Figure 8:**
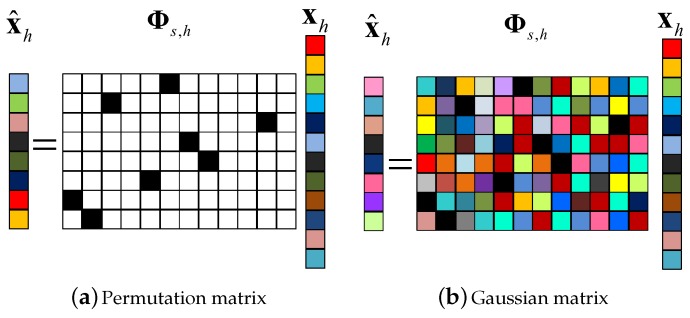
The active CS process at each array sensor using a projection matrix constructed from a permutation or Gaussian matrix.

To eliminate the adverse effects of block data loss on DOA estimation in WSAN, besides the passive CS process, we introduce an active CS process at each array sensor to form the double-layer CS framework. At the *h*-th array sensor, instead of directly transmitting the raw data vector $x_{h}$, we perform a dimension-reduced projection on it before its elements are assembled into packets for transmission, as shown in Figure 8. The projection matrix can be a Gaussian matrix, which makes each of the after-projection data samples a weighted average of the raw data samples. In this way, the transmitted data samples all contain global information of the impinging signals. Besides, a permutation matrix is also competent. By using this matrix, we can rearrange the raw data samples so that the originally adjacent ones will not be assembled into the same data packet. The projection operation using either of the matrices mentioned above can get rid of the hazards of block data loss. The projection matrix is generated in advance and stored in the ROM of the array sensors. For a favorable scalability, an identical projection matrix is chosen for all array sensors. To compensate for the energy consumption generated by the projection operation, we make it dimension-reduced to lower the data volume. Therefore, the active CS projection matrix can be constructed by selecting $M_{s}\left(M_{s}<N\right)$ random rows from an N×N Gaussian or permutation matrix. After the completion of projection at each array sensor, the newly-generated data samples are assembled into data packets according to the packet size and then transmitted to the FC over lossy wireless links. Note that under the double-layer CS framework, the equivalent measurement matrix is denoted by $A=\Phi_{r}\Phi_{s}\Psi$. Likewise, we present the histograms of the absolute off-diagonal elements of the corresponding Gram matrix (taking the case where $\Phi_{s}$ is a permutation matrix as an example) under different packet sizes. As shown in Figure 7b, the distribution of entries almost stays unchanged since the effect of the projection operation (active CS process) is to reduce the number of very correlated columns in A. Furthermore, the values of mutual coherence for the single-layer CS and double-layer CS framework under different packet sizes are provided in Table 2. The results are self-explanatory, the values of $\mu \left(A\right)$ and $\mu_{av}\left(A\right)$ keep rising with the increase of packet size under the single-layer CS framework. However, under the double-layer CS framework, the values remain low regardless of the packet size, which promises a superior DOA estimation performance.

### 3.2. DOA Estimation by DCS-DOA

Under the double-layer CS framework, the received data samples from each array sensor can be regarded as a measurement vector, and we have *H* measurement vectors in total at the FC. For DCS-DOA, the DOA estimation results are obtained in two stages. In the first stage, the raw sparse vectors are required to be recovered from the received data samples by sparsity-based techniques. In the second stage, DOA estimation is implemented according to the recovered sparse vectors. The details of the two stages are presented respectively in the following two subsections.

#### 3.2.1. Signal Recovery

The active CS projection matrix at each array sensor is generated in advance and stored in the ROM, as well as at the FC. Moreover, as the sending sequence number of each data packet can be included in the packet header, the identities of the received data packets are known by the FC. For the *h*-th array sensor, taking the passive CS process, the active CS process and the sparse representation in the frequency domain into consideration simultaneously, we have:

(7) $$y_{h}=\Phi_{r,h}\Phi_{s,h}x_{h}=\Phi_{r,h}\Phi_{s,h}\Psi \alpha_{h}=\Phi_{h}\Psi \alpha_{h}$$

where $y_{h}$ is the received data vector (the measurement vector), $\Phi_{r,h}$ is the passive CS measurement matrix modeling the packet loss, $\Phi_{s,h}$ is the active CS projection matrix adopted at the *h*-th array sensor, Ψ is the N×N inverse discrete Fourier transform (IDFT) matrix and $\alpha_{h}$ is the sparse vector in the frequency domain. For the *h*-th array sensor, the double-layer CS measurement matrix is denoted by $\Phi_{h}$, and $\Phi_{h}=\Phi_{r,h}\Phi_{s,h}$. Stacking all of the measurement vectors of the *H* array sensors together, we have:

(8) $$\left[\begin{array}{c} y_{1}\\ y_{2}\\ \vdots \\ y_{H} \end{array}\right]=\left[\begin{array}{cccc} \Phi_{1}\Psi & & & \\ & \Phi_{2}\Psi & & \\ & & \ddots & \\ & & & \Phi_{H}\Psi \end{array}\right]\left[\begin{array}{c} \alpha_{1}\\ \alpha_{2}\\ \vdots \\ \alpha_{H} \end{array}\right]$$

or in a concise form:

(9) y=Θα

where $y={\left[y_{1}^{T},\ldots ,y_{H}^{T}\right]}^{T}$ is the joint measurement vector, $\Theta =\mathrm{diag}\left\{\Phi_{1}\Psi ,\Phi_{2}\Psi ,\ldots ,\Phi_{H}\Psi \right\}$ is the joint measurement matrix of *H* array sensors and $\alpha ={\left[\alpha_{1}^{T},\ldots ,\alpha_{H}^{T}\right]}^{T}$ is the joint sparse vector. To implement DOA estimation, the sparse vector at each array sensor needs to be recovered. Considering that $\alpha_{h},1\leq h\leq H$ share the same sparse structure under the assumption that signals of interest are composed of identical harmonics, the sparse vector reconstruction, given Equation (9), can be realized based on the concept of group-sparsity [26]. Define a matrix $\beta =\left[\alpha_{1},\alpha_{2},\ldots ,\alpha_{H}\right]$, with $\beta_{k}$ denoting the *k*-th row of β, and $\bar{\alpha }={\left[{∥\beta_{1}∥}_{2},{∥\beta_{2}∥}_{2},\ldots ,{∥\beta_{N}∥}_{2}\right]}^{T}$; then, $\alpha_{h},1\leq h\leq H$ can be recovered by solving the following group sparsity-based $l_{1}$-norm minimization problem:

(10) $$\begin{array}{c} \min\;\;\;\;\;\;\;\;\;\;\;\;\;\;\;\;\;\;\;\;\;\;\;\;\;\;\;\;\;{∥\bar{\alpha }∥}_{1}\\ \mathrm{subject}\;\;\;\;\mathrm{to}\;\;\;\;\;\;y=\Theta \alpha \end{array}$$

which is representable in a second order cone programming (SOCP) frame and solved using the SeDuMi toolbox [27].

#### 3.2.2. DOA Estimation

After recovering the sparse vector at each array sensor, DOA estimation can be realized using various algorithms, such as the MUSIC technique and the maximum likelihood estimator. In this paper, a sparse signal reconstruction perspective is adopted for better estimation accuracy and robustness to noise. We denote the recovered sparse vector at the *h*-th array sensor by ${\tilde{\alpha }}_{h},h=1,\ldots ,H$. Now, the array output data are already in the frequency domain, so we can directly perform DOA estimation without the need for DFT conducted in the common wideband DOA estimation process. Assume that ${\tilde{\alpha }}_{h}$ contains $K\left(K\ll N\right)$ nonzero elements; we only need to consider the specific *K* nonzero frequency sub-bands. First, we investigate the DOA estimation problem at a single frequency sub-band (denoted by $f_{k}$, chosen from the *K* nonzero frequency components), and the array output vector at $f_{k}$ can be denoted by $\tilde{\alpha }\left[f_{k}\right]={\left[{\tilde{\alpha }}_{1}\left[f_{k}\right],\ldots ,{\tilde{\alpha }}_{H}\left[f_{k}\right]\right]}^{T}$. Similarly, the noise vector for the sensor array at the frequency sub-band $f_{k}$ is denoted by $n\left[f_{k}\right]={\left[n_{1}\left[f_{k}\right],\ldots ,n_{H}\left[f_{k}\right]\right]}^{T}$. Using a search grid of *Q* elements, with each of them indicating a potential source signal at the corresponding incident angle, the array output vector at the frequency sub-band $f_{k}$ can be formulated as follows:

(11) $$\tilde{\alpha }\left[f_{k}\right]=\tilde{A}\left(f_{k},\tilde{\theta }\right)\tilde{s}\left[f_{k}\right]+n\left[f_{k}\right]$$

where $\tilde{A}\left(f_{k},\tilde{\theta }\right)=\left[\tilde{a}\left(f_{k},{\tilde{\theta }}_{1}\right),\ldots ,\tilde{a}\left(f_{k},{\tilde{\theta }}_{Q}\right)\right]$ is the array manifold matrix corresponding to the virtual source signal vector $\tilde{s}\left[f_{k}\right]={\left[\tilde{s}\left[f_{k},{\tilde{\theta }}_{1}\right],\tilde{s}\left[f_{k},{\tilde{\theta }}_{2}\right],\ldots ,\tilde{s}\left[f_{k},{\tilde{\theta }}_{Q}\right]\right]}^{T}$, and $\tilde{a}\left(f_{k},{\tilde{\theta }}_{q}\right)$ is the steering vector related to ${\tilde{\theta }}_{q}$. The sampling grids of all potential directions are denoted by a vector $\tilde{\theta }={\left[{\tilde{\theta }}_{1},\ldots ,{\tilde{\theta }}_{Q}\right]}^{T}$. As the sampling grid is sufficiently fine, the number of nonzero elements in $\tilde{s}\left[f_{k}\right]$ is much smaller than *Q*. That is to say, $\tilde{s}\left[f_{k}\right]$ is sparse, and the indexes of the nonzero elements indicate the DOAs of the actual sources. A sparse signal reconstruction perspective for DOA estimation was proposed in [3], and it can be applied to a single frequency sub-band in the wideband case directly, so the aforementioned DOA estimation problem can be resolved by solving the optimization problem below:

(12) $$\begin{array}{c} \min\;\;\;\;\;\;\;\;\;\;\;\;\;\;\;\;\;\;\;\;\;\;\;\;\;\;\;\;\;\;{∥\tilde{s}\left[f_{k}\right]∥}_{1}\\ \mathrm{subject}\;\;\;\;\mathrm{to}\;\;\;\;\;{∥\tilde{\alpha }\left[f_{k}\right]-\tilde{A}\left(f_{k},\tilde{\theta }\right)\tilde{s}\left[f_{k}\right]∥}_{2}\leq \varepsilon_{1} \end{array}$$

where $\varepsilon_{1}$ is the user-specified error bound, and the $l_{1}$-norm and the $l_{2}$-norm represent the sparsity penalty and the residual error, respectively.

As the DOAs corresponding to the $K\left(K\ll N\right)$ nonzero frequency components share the same spatial sparse pattern, we can estimate the DOAs of wideband source signals based on the concept of group-sparsity. Assume that the frequencies of the *K* nonzero sub-bands are denoted by $f_{1},\ldots ,f_{K}$. Now, we construct two matrices: $B=\mathrm{diag}\left\{\tilde{A}\left(f_{1},\tilde{\theta }\right),\tilde{A}\left(f_{2},\tilde{\theta }\right),\ldots ,\tilde{A}\left(f_{K},\tilde{\theta }\right)\right\}$, $\tilde{S}=\left[\tilde{s}\left[f_{1}\right],\tilde{s}\left[f_{2}\right],\ldots ,\tilde{s}\left[f_{K}\right]\right]$. Combining all of the *K* nonzero frequency sub-bands, we have:

(13) $$\tilde{\alpha }=B\tilde{s}+n$$

where $\tilde{\alpha }={\left[{\tilde{\alpha }}^{H}\left[f_{1}\right],\ldots ,{\tilde{\alpha }}^{H}\left[f_{K}\right]\right]}^{H}$ is the joint array output vector covering the *K* sub-bands, and $\tilde{s}=\mathrm{vec}\left(\tilde{S}\right)$ is a KQ×1 joint sparse indicator vector by vectorizing $\tilde{S}$. Additionally, $n={\left[n^{H}\left[f_{1}\right],\ldots ,n^{H}\left[f_{K}\right]\right]}^{H}$ is the joint noise vector.

We use the row vector $s_{q}$, q=1,…,Q, to represent the *q*-th row of $\tilde{S}$. Based on the $l_{2}$-norm of $s_{q}$, we can formulate a new Q×1 column vector $\hat{s}$ as $\hat{s}={\left[{∥s_{1}∥}_{2},{∥s_{2}∥}_{2},\ldots ,{∥s_{Q}∥}_{2}\right]}^{T}$. Ultimately, the DOA estimation problem of wideband source signals based on group-sparsity is formulated as follows:

(14) $$\begin{array}{c} \min\;\;\;\;\;\;\;\;\;\;\;\;\;\;\;\;\;\;\;\;\;\;\;\;\;\;\;\;\;{∥\hat{s}∥}_{1}\\ \mathrm{subject}\;\;\;\;\mathrm{to}\;\;\;\;{∥\tilde{\alpha }-B\tilde{s}∥}_{2}\leq \varepsilon_{2} \end{array}$$

where $\varepsilon_{2}$ is the user-specified error bound, and the problem is representable in an SOCP frame and solved using the SeDuMi toolbox [27]. The indexes of the nonzero elements in $\hat{s}$ indicate the wideband DOA estimation results.

### 3.3. Improved DOA Estimation by DCS-DDOA

To avoid the error propagation from signal recovery to DOA estimation in DCS-DOA, we develop a new scheme DCS-DDOA, where a joint frequency and spatial domain sparse representation of the sensor array data is constructed and leveraged. Therefore, we can directly conduct DOA estimation at the FC based on the received data samples without the need for signal recovery.

#### 3.3.1. Joint Sparse Representation

Suppose an *N*-point DFT has been performed on the raw data vector at each array sensor, i.e., $x_{h}=\Psi \alpha_{h},h=1,\ldots ,H$. As we plan to skip the step of signal recovery at the FC, the locations of the nonzero elements in $\alpha_{h}$ are unknown. Therefore, we need to deal with all of the *N* frequency sub-bands. In a similar fashion, the array output vector at the *n*-th frequency sub-band $\bar{\alpha }\left[n\right]$ is presented as:

(15) $$\bar{\alpha }\left[n\right]=\bar{A}\left(n,\bar{\theta }\right)\bar{s}\left[n\right]+w\left[n\right]$$

where $\bar{\alpha }\left[n\right]={\left[{\bar{\alpha }}_{1}\left[n\right],\ldots ,{\bar{\alpha }}_{H}\left[n\right]\right]}^{T}$, $\bar{A}\left(n,\bar{\theta }\right)=\left[\bar{a}\left(n,{\bar{\theta }}_{1}\right),\ldots ,\bar{a}\left(n,{\bar{\theta }}_{Q}\right)\right]$ is the array manifold matrix corresponding to the virtual source signal vector $\bar{s}\left[n\right]$ and $\bar{a}\left(n,{\bar{\theta }}_{q}\right)$ is the steering vector related to ${\bar{\theta }}_{q}$. The sampling grid of all potential directions is denoted by $\bar{\theta }={\left[{\bar{\theta }}_{1},\ldots ,{\bar{\theta }}_{Q}\right]}^{T}$, and $w\left[n\right]$ is the noise vector.

Spontaneously, we can derive the joint array output vector across the *N* frequency sub-bands as follows:

(16) $$\bar{\alpha }=\bar{B}\bar{s}+w$$

where $\bar{\alpha }={\left[{\bar{\alpha }}^{H}\left[0\right],{\bar{\alpha }}^{H}\left[1\right],\ldots ,{\bar{\alpha }}^{H}\left[N-1\right]\right]}^{H}$, $\bar{s}={\left[{\bar{s}}^{H}\left[0\right],{\bar{s}}^{H}\left[1\right],\ldots ,{\bar{s}}^{H}\left[N-1\right]\right]}^{H}$ is the joint sparse indicator vector, $w={\left[w^{H}\left[0\right],w^{H}\left[1\right],\ldots ,w^{H}\left[N-1\right]\right]}^{H}$ is the joint noise vector and $\bar{B}=\mathrm{diag}\left\{\bar{A}\left(0,\bar{\theta }\right),\bar{A}\left(1,\bar{\theta }\right),\ldots ,\bar{A}\left(N-1,\bar{\theta }\right)\right\}$ is the joint array manifold matrix, with a dimension of HN×QN.

Let us define a H×N matrix $D=\left[\bar{\alpha }\left[0\right],\bar{\alpha }\left[1\right],\ldots ,\bar{\alpha }\left[N-1\right]\right]$. Observe that D can also be represented as $D={\left[\alpha_{1},\alpha_{2},\ldots ,\alpha_{H}\right]}^{T}$. Obviously, $\bar{\alpha }=\mathrm{vec}\left(D\right)$ and $\alpha =\mathrm{vec}\left(D^{T}\right)$, so the elements in α and $\bar{\alpha }$ are just the identical elements from the matrix D. In this way, we can construct a permutation matrix P such that:

(17) $$\alpha =P\bar{\alpha }$$

where P is utilized to adjust the locations of the elements in D, mapping $\bar{\alpha }$ onto α, which can be easily obtained. Equation (17) relates α, which emphasizes the sparsity in the frequency domain, to $\bar{\alpha }$, which is the virtual array output vector corresponding to the sparse sources in the spatial domain. In other words, a joint frequency and spatial domain sparse representation of the sensor array data, which will be used for DOA estimation in the next step, is exhibited through the elements in matrix D.

#### 3.3.2. Direct DOA Estimation

Leveraging the joint frequency and spatial domain sparse representation of the sensor array data, DOA estimation can be directly implemented at the FC according to the received data samples. Substituting Equations (16) and (17) into Equation (9), we can have:

(18) $$\begin{array}{c} y=\Theta \alpha =\Theta P\bar{\alpha }=\Theta P\left(\bar{B}\bar{s}+w\right)=\Gamma \bar{s}+\bar{w} \end{array}$$

where $\bar{w}=\Theta \mathrm{Pw}$ is the subsampled white Gaussian noise at the FC, and $\Gamma =\Theta P\bar{B}$ is the joint sparse representation matrix with a dimension of HM×NQ, combining the frequency sparsity in Θ and the spatial sparsity in $\bar{B}$. The structure of Γ is shown as follows:

(19) $$\Gamma =\left[\begin{array}{cccc} \Theta_{1,1}{\bar{a}}_{1}\left(0\right) & \Theta_{1,2}{\bar{a}}_{1}\left(1\right) & \cdots & \Theta_{1,N}{\bar{a}}_{1}\left(N-1\right)\\ \Theta_{2,1}{\bar{a}}_{2}\left(0\right) & \Theta_{2,2}{\bar{a}}_{2}\left(1\right) & \cdots & \Theta_{2,N}{\bar{a}}_{2}\left(N-1\right)\\ \vdots & \vdots & \vdots & \vdots \\ \Theta_{H,1}{\bar{a}}_{H}\left(0\right) & \Theta_{H,2}{\bar{a}}_{H}\left(1\right) & \cdots & \Theta_{H,N}{\bar{a}}_{H}\left(N-1\right) \end{array}\right]$$

where $\Theta_{h,n}$ is the *n*-th column of $\Theta_{h}$, i.e., $\Phi_{r,h}\Phi_{s,h}\Psi$ and ${\bar{a}}_{h}\left(n\right)$ is the *h*-th row of $\bar{A}\left(n\right)$. Now, we can reconstruct the joint sparse indicator vector $\bar{s}$ from the joint measurement vector y to obtain the DOA estimation results without the need for recovering the sparse vectors $\alpha_{h},1\leq h\leq H$.

Define a matrix $\bar{S}=\left[\bar{s}\left[0\right],\bar{s}\left[1\right],\ldots ,\bar{s}\left[N-1\right]\right]$, with ${\bar{s}}_{q},q=1,\ldots ,Q$ denoting the *q*-th row vector of $\bar{S}$. On the basis of the mentioned representations above and the concept of group sparsity, we can formulate the DOA estimation problem as the following constrained $l_{1}$-norm minimization problem:

(20) $$\begin{array}{c} \min\;\;\;\;\;\;\;\;\;\;\;\;\;\;\;\;\;\;\;\;\;\;\;\;\;\;\;\;\;\;\;\;\;\;{∥\gamma ∥}_{1}\\ \mathrm{subject}\;\;\;\;\mathrm{to}\;\;\;\;\;\;\;\;\;{∥y-\Gamma \bar{s}∥}_{2}\leq \varepsilon_{3} \end{array}$$

where $\gamma ={\left[{∥{\bar{s}}_{1}∥}_{2},{∥{\bar{s}}_{2}∥}_{2},\ldots ,{∥{\bar{s}}_{Q}∥}_{2}\right]}^{T}$ is the sparse indicator vector and $\varepsilon_{3}$ is the user-specified error bound. In the same way, the problem in Equation (20) is solved using the SOCP approach by virtue of the SeDuMi toolbox. Finally, the DOA estimation results of the wideband source signals are spontaneously deduced according to the sparse solution to γ.

Different from the two-stage DCS-DOA, the DCS-DDOA can desirably exploit the joint sparsity in the frequency and spatial domain. In this integrated approach, it can subtly avoid the error propagation problem existing in the two-stage DCS-DOA. More specifically, the formulated problem is capable of directly obtaining the DOA estimation results, as shown in (20). As a consequence, the DOA estimation performance can be further improved.

## 4. Experimental Results

In this section, a series of numerical simulation results is presented to illustrate the DOA estimation performance achieved by DCS-DOA and DCS-DDOA. We use CS-DOA as a baseline, where DOA estimation is implemented under the single-layer CS framework. The simulations are performed using MATLAB2010b running on an Intel Core i5-4460, 3.20-GHz processor with 12 GB memory, under Windows 7.

### 4.1. Simulation Settings

The signals imitating passive acoustic targets are synthesized using four dominant frequencies at 320 Hz, 480 Hz, 640 Hz and 800 Hz. In this section, we assume that the acoustic signal is spread in the air and that the propagation speed is 340 m/s (the following mentioned guidelines on how to design the array geometry can be easily extended to other scenarios, where the acoustic signal may have a different propagation speed). Therefore, the minimum wavelength of the impinging signals is 0.425 m. The received acoustic signals are contaminated with zero-mean, white Gaussian noise. Additionally, the variance of noise can be altered to obtain different SNR values. The sampling rate is set to 4096 Hz, which is much more than twice the highest frequency of interest, to avoid the frequency aliasing effect. We consider a WSAN consisting of a six-element ULA (note that the results in the paper can be easily extended to non-uniform linear arrays) and one FC, where the inter-element spacing in the ULA is set 0.2 m, which is smaller than half of the minimum wavelength of the impinging signals to avoid angle ambiguity effects. Array sensors are connected to the FC through lossy wireless links. For each array sensor, *N* = 512 data samples will be collected to implement one DOA estimation process. The acquired data samples are assembled into data packets for transmission to the FC over lossy wireless links. For the ease of simulation and analysis, we ignore the overhead produced by the packet header and packet footer, and the data packet size is set to 1, 8, 16, 32 and 64, indicating the number of data samples grouped into one data packet. The incident angles (DOAs) of the acoustic signals are constrained within the range from −$90^{\circ }$–$90^{\circ }$, and this angle range is divided into 360 search grids, with $0.5^{\circ }$ spacing. The number of data samples received from each array sensor at the FC is denoted by *M*. In the following simulations, three evaluation criteria are used to describe and compare the performance of CS-DOA, DCS-DOA and DCS-DDOA. The reconstruction error is defined as η=∑h=1Hx˜h−xh22∑h=1Hx˜h−xh22∑h=1Hxh22∑h=1Hxh22. Following a similar work [28], which takes into account both the errors in estimating the actual source number and the corresponding DOAs, the root mean square error is defined as: RMSE=11L∑l=1LRMSElL∑l=1LRMSEl, and $\mathrm{RMS}E^{\left(l\right)}$ is defined as: when ${\tilde{K}}^{\left(l\right)}\leq \tilde{K}$,

$$\mathrm{RMS}E^{\left(l\right)}=\sqrt{\frac{\sum_{k=1}^{{\tilde{K}}^{\left(l\right)}}{\left|\theta_{k}-{\tilde{\theta }}_{k}^{\left(l\right)}\right|}^{2}+\left|\tilde{K}-{\tilde{K}}^{\left(l\right)}\right|{\left(\Delta \theta_{\max}\right)}^{2}}{\tilde{K}}}$$

when ${\tilde{K}}^{\left(l\right)}>\tilde{K}$,

$$\mathrm{RMS}E^{\left(l\right)}=\sqrt{\frac{\sum_{k=1}^{\tilde{K}}{\left|\theta_{k}-{\tilde{\theta }}_{k}^{\left(l\right)}\right|}^{2}+\sum_{j=\tilde{K}+1}^{{\tilde{K}}^{\left(l\right)}}{\left|{\tilde{\theta }}_{j}^{\left(l\right)}-{\bar{\theta }}_{j}^{\left(l\right)}\right|}^{2}}{\tilde{K}}}$$

where $\tilde{K}$ is the number of actual sources, ${\tilde{K}}^{\left(l\right)}$ is the number of estimated sources of the *l*-th trial, *L* is the number of detection trials and ${\bar{\theta }}_{j}^{\left(l\right)}=\arg\;\;\;\left\{\min_{\theta_{k},k=1,\ldots ,\tilde{K}}\left|\theta_{k}-{\tilde{\theta }}_{j}^{\left(l\right)}\right|\right\}$, $\Delta \theta_{\max}$ is a penalty term, which is equal to the maximum admissible localization error (i.e., $\Delta \theta_{\max}=180^{\circ }$). For completeness, the detection frequency is defined as the percentage of successful detections (i.e., $\tilde{K}={\tilde{K}}^{\left(l\right)}$) in *L* independent trials.

### 4.2. Performance Analysis for DCS-DOA

**Figure 9:**
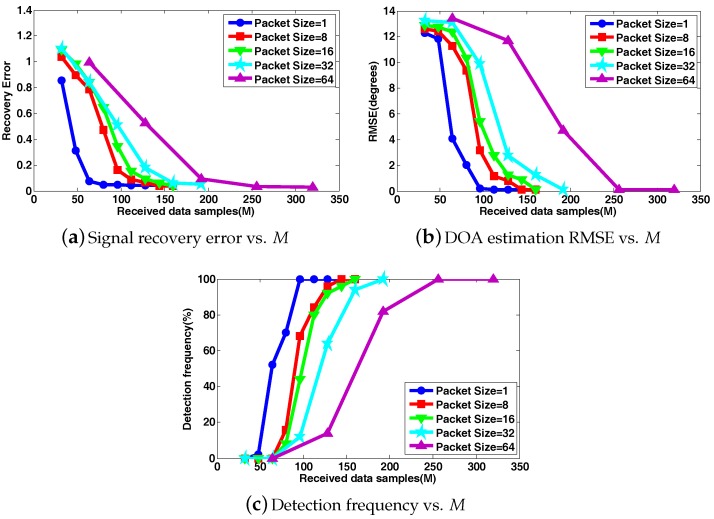
The signal recovery error, DOA estimation RMSE and detection frequency versus the number of received data samples *M* under different packet sizes when projection (active CS) is not introduced at each array sensor.

To highlight the adverse effects of block data loss on DOA estimation in WSAN, we first present the DOA estimation behavior of CS-DOA, where the projection operation (the active CS process) is not adopted at each array sensor, while the passive CS process and DOA estimation are identical to those in DCS-DOA. In this set of simulations, two ($\tilde{K}$ = 2) stationary acoustic sources from directions $\theta_{1}=-60^{\circ }$, $\theta_{2}=30^{\circ }$ are impinging on the ULA. The input SNR is set to 10 dB. The group LASSO algorithm in [26] is leveraged to solve the optimization problems. Five hundred independent trials are performed to provide the averaged results. As shown in Figure 9a–c, all of the metrics of signal recovery error, DOA estimation RMSE and detection frequency become worse when a larger packet size is chosen. In other words, the FC will need to receive more data samples to provide a satisfactory DOA estimation result with a larger packet size, which explicitly reveals the hazards of block data loss.

**Figure 10:**
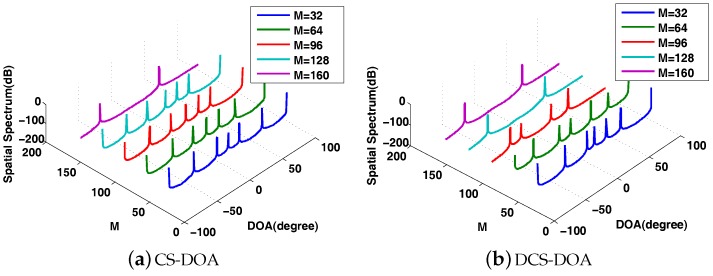
The comparison of DOA spatial spectrum between CS-DOA and DCS-DOA with packet size = 32.

**Figure 11:**
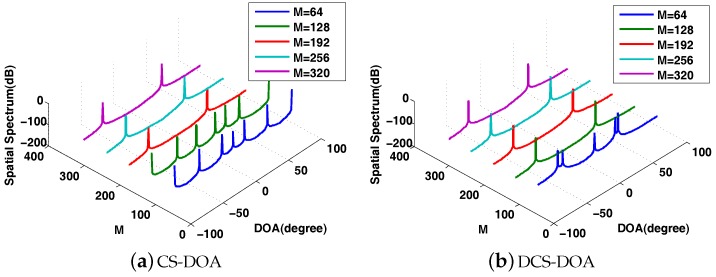
The comparison of DOA spatial spectrum between CS-DOA and DCS-DOA with packet size = 64.

In DCS-DOA, a projection operation (active CS process) is introduced at each array sensor. To compensate for the energy consumption generated by the projection operation, we make the projection matrix dimension-reduced; thus, the energy consumption for transmission is reduced. A proper compression ratio of the active CS process is determined through trial and error to make sure that an accurate DOA estimation result can be obtained even when there is a somewhat high packet loss rate. We present the DOA spatial spectrum to make an intuitional comparison between CS-DOA and DCS-DOA. According to the DOA spatial spectrum, DCS-DOA shows a better DOA estimation behavior than CS-DOA. As shown in Figure 10, when the packet size is set to 32, the FC needs 160 data samples from each array sensor to obtain an unambiguous DOA spectrum in CS-DOA, while the number is just 128 in DCS-DOA. When the packet size is 64, as shown in Figure 11, 128 data samples are still enough for DCS-DOA to provide an acceptable DOA estimation result, while CS-DOA fails until the number reaches 192.

**Figure 12:**
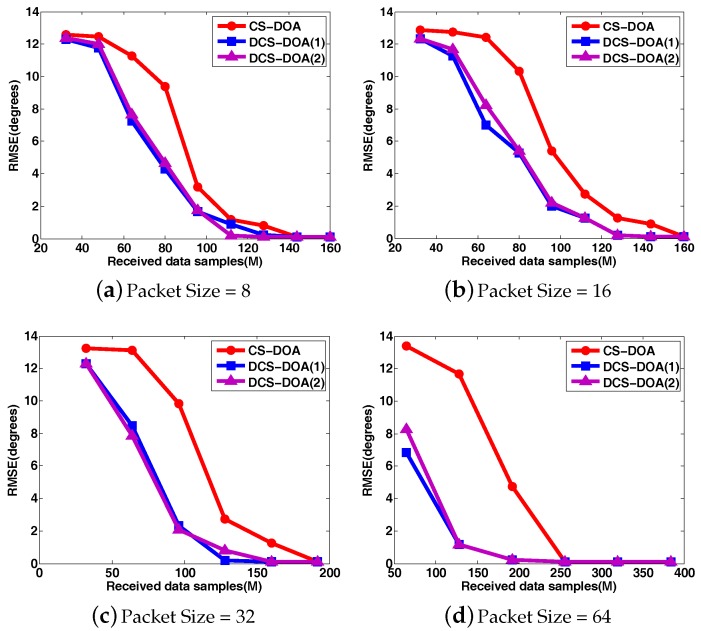
The comparison of DOA RMSE between CS-DOA and DCS-DOA under different packet sizes.

**Figure 13:**
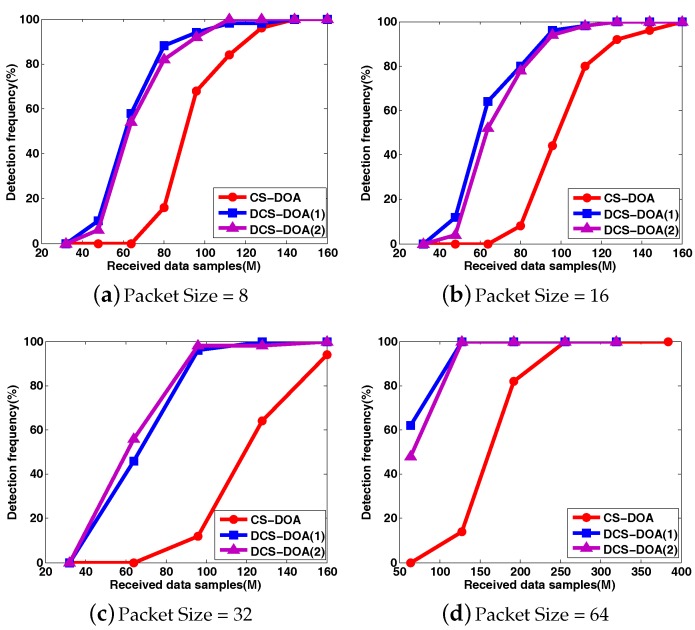
The comparison of detection frequency between CS-DOA and DCS-DOA under different packet sizes.

To clearly compare the DOA estimation performance for CS-DOA and DCS-DOA under different packet sizes, the DOA RMSE and the detection frequency results are shown in Figure 12 and Figure 13, where each point is based on an average of the results obtained from 500 independent simulation runs. DCS-DOA is denoted by DCS-DOA(1) if the projection matrix at each array sensor is a dimension-reduced permutation matrix, and it is denoted by DCS-DOA(2) if the projection matrix is constructed from a random Gaussian matrix.

**Figure 14:**
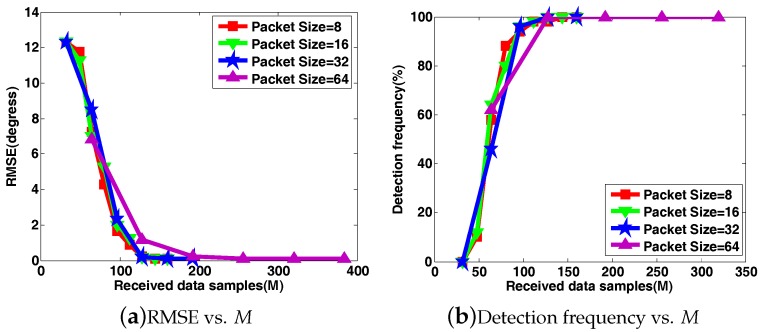
The DOA RMSE and detection frequency for DCS-DOA versus *M* under different packet sizes.

According to the simulation results, whatever the packet size, the DOA estimation performance achieved by DCS-DOA has obvious advantages over CS-DOA. Additionally, the DOA estimation RMSE and detection frequency for DCS-DOA is no longer affected by block data loss, as shown in Figure 14. Therefore, we can exploit a larger packet size to achieve a higher data transmission efficiency. The performances achieved by DCS-DOA(1) and DCS-DOA(2) are verified to be very close to each other. Considering that the storage, fetch and processing of a permutation matrix is simpler and less energy-consuming, the projection matrix is usually constructed from a permutation matrix. In the next simulations, we use DCS-DOA(1) as a representative, simply denoted by DCS-DOA.

### 4.3. Performance Analysis for DCS-DDOA

**Figure 15:**
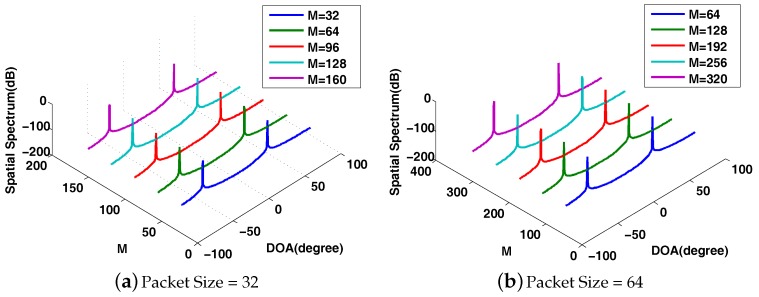
The DOA spatial spectrum for DCS-DDOA with the packet size being 32 and 64.

**Figure 16:**
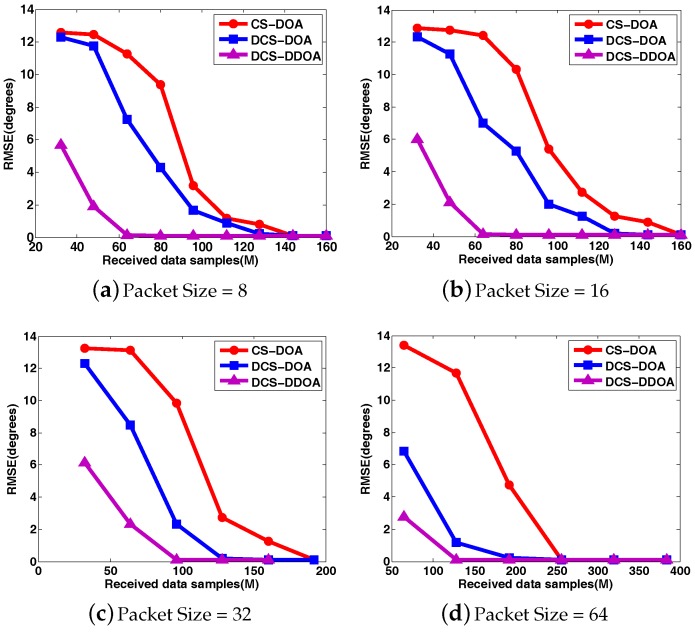
The comparison of DOA estimation RMSE among CS-DOA, DCS-DOA, DCS-DDOA under different packet sizes.

**Figure 17:**
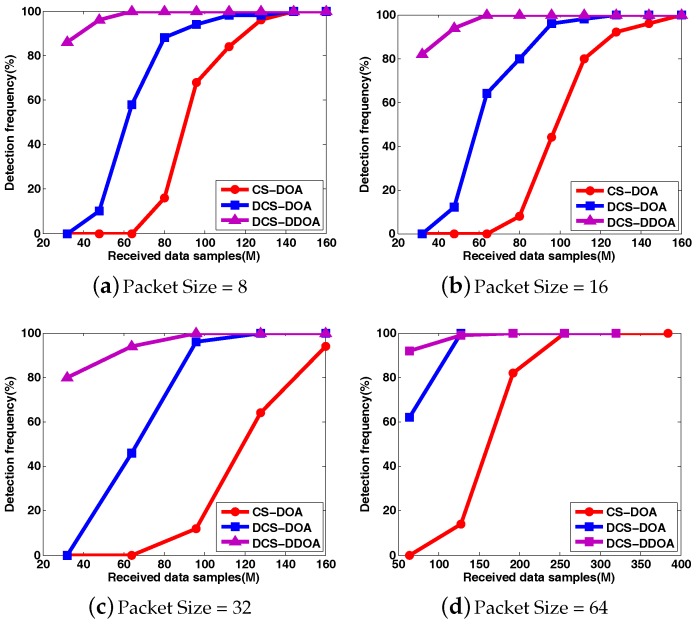
The comparison of detection frequency among CS-DOA, DCS-DOA, DCS-DDOA under different packet sizes.

Similarly, we first present the behavior of DOA estimation for DCS-DDOA in a direct way. Assume that there are two fixed sources with incident angles $\theta_{1}=-60^{\circ }$ and $\theta_{2}=30^{\circ }$. The input SNR is 10 dB. The DOA spatial spectrum corresponding to different packet sizes is shown in Figure 15. When the packet size is 32, DCS-DDOA provides a clear DOA spectrum with a high probability as long as the FC receives 32 data samples from each array sensor, and the value becomes 64 when the packet size is increased to 64. Leveraging the joint sparsity in the frequency and spatial domain, DCS-DDOA is able to directly perform DOA estimation, skipping the signal recovery stage. Therefore, the error propagation problem is avoided in DCS-DDOA, which yields a further improved DOA estimation performance, as shown in Figure 16 and Figure 17.

**Figure 18:**
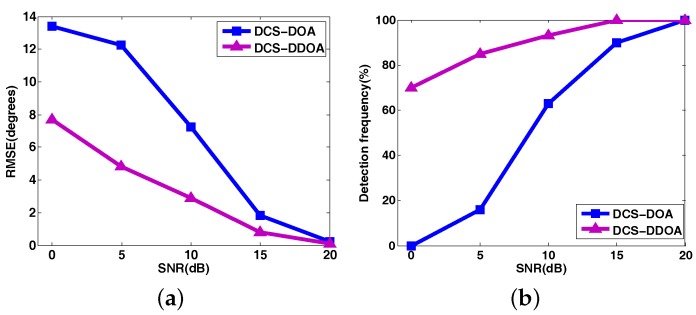
The comparison of DOA estimation RMSE and detection frequency between DCS-DOA and DCS-DDOA under a varying SNR with the packet size being 64 and *M* being 64.

**Figure 19:**
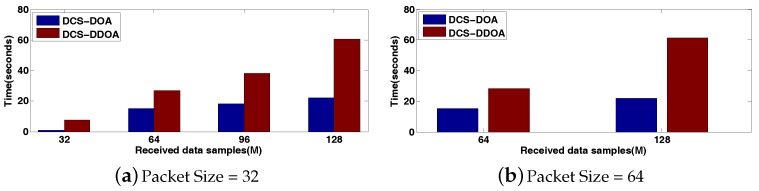
The computation time spent on one DOA estimate for DCS-DOA and DCS-DDOA.

Furthermore, we make a comparison between DCS-DOA and DCS-DDOA under a varying SNR, given that the packet size is 64 and the number of received data samples from each array sensor is *M* = 64. The results are shown in Figure 18, where each point is based on an average of 500 independent trials. Obviously, the DOA estimation performance of DCS-DDOA is always better than DCS-DOA regardless of the noise level. This results from the avoidance of the error propagation and the exploitation of a joint sparse representation. However, it costs something else to obtain such a satisfactory DOA estimation behavior. Now, we conduct a statistical analysis to get the computation time spent on one DOA estimate for the two techniques. In accordance with previous simulation results, when the packet size is 64, the behavior of DOA estimation is nearly perfect for both methods when *M* reaches 128, so we just explore the computation time in cases where *M* = 64, 128. By the same token, the cases of *M* = 32, 64, 96, 128 are investigated when the packet size is 32. The results are shown in Figure 19, which is obtained based on an average of 500 independent trials.

As depicted in Figure 19, it takes much more time for DCS-DDOA to complete one DOA estimation process. Thus, it would be inapplicable in some scenarios where there is an urgent need for real-time target localization and tracking. In practice, if the quality of wireless links can be estimated in real time, we can combine it with the application requirements to decide which approach to adopt. If the link quality is sufficiently good, we will choose DCS-DOA for a faster DOA estimation process. Otherwise, we need to utilize DCS-DDOA to ensure the DOA estimation accuracy.

## 5. Conclusions

In this paper, we investigate the adverse effects of block data loss on DOA estimation in WSAN. To eliminate the hazards of block data loss and realize high-accuracy and efficient DOA estimation, we propose a double-layer compressive sensing framework, where the random packet loss during transmission is modeled as a passive CS process, while the dimension-reduced random projection at each array sensor is modeled as an active CS process. Under the double-layer CS framework, DOA estimation can be implemented using two techniques, DCS-DOA and DCS-DDOA. In DCS-DOA, the FC will first recover the raw sparse vectors and then perform DOA estimation based on them. To avoid the error propagation from signal recovery to DOA estimation, DCS-DDOA is proposed. Leveraging a joint frequency and spatial domain sparse representation of the sensor array data, the FC can directly obtain the DOA estimation results according to the received data, skipping the phase of signal recovery. Extensive simulations demonstrate that the double-layer CS framework can be immune to block data loss and yield a satisfactory DOA estimation performance in WSAN. Furthermore, a comparison is made between DCS-DOA and DCS-DDOA to show their merits and deficiencies.

## Figures and Tables

**Table 1:** Notations and explanations. FC, fusion center.

Notations	Explanations
$x_{h}$	raw data samples at the *h*-th array sensor
Ψ	Fourier sparsifying dictionary
$\alpha_{h}$	sparse representation of $x_{h}$ under the basis Ψ
$\Phi_{s}$	the active CS projection matrix adopted at each array sensor
${\hat{x}}_{h}$	newly-generated data samples after a projection on $x_{h}$
$\Phi_{r}$	the passive CS measurement matrix modeling the packet loss
$y_{h}$	received data samples at the FC from the *h*-th array sensor
${\tilde{x}}_{h}$	recovered data samples for the *h*-th array sensor at the FC

**Table 2:** The mutual coherence of the equivalent measurement matrix under the single-layer CS and double-layer CS framework when *N* = 512 and *M* = 64.

	Single-Layer CS		Double-Layer CS	
**Packet Size**	$\mu \left(A\right)$	$\mu_{\mathrm{av}}\left(A\right)$	$\mu \left(A\right)$	$\mu_{\mathrm{av}}\left(A\right)$
1	0.2859	0.2297	0.2825	0.2306
8	0.5911	0.3328	0.2905	0.2269
16	0.7489	0.4077	0.2920	0.2311
32	0.8866	0.5118	0.2927	0.2296
64	0.9745	0.5616	0.2929	0.2308

## References

[1] Krim H., Viberg M. (1996). Two decades of array signal processing research: the parametric approach. IEEE Signal Process. Mag..

[2] Nagata Y., Fujioka T., Abe M. (2007). Two-Dimensional DOA Estimation of Sound Sources Based on Weighted Wiener Gain Exploiting Two-Directional Microphones. IEEE Trans. Audio Speech Lang. Process..

[3] Malioutov D., Cetin M., Willsky A.S. (2003). A sparse signal reconstruction perspective for source localization with sensor arrays. IEEE Trans. Signal Process..

[4] Shao H.J., Zhang X.P., Wang Z. Novel closed-form auxiliary variables based algorithms for sensor node localization using AOA. Proceedings of the 2014 IEEE International Conference on Acoustics, Speech and Signal Processing (ICASSP 2014).

[5] Shao H.J., Zhang X.P., Wang Z. (2014). Efficient Closed-Form Algorithms for AOA Based Self-Localization of Sensor Nodes Using Auxiliary Variables. IEEE Trans. Signal Process..

[6] Winder A.A. II. (1975). Sonar system technology. IEEE Trans. Sonics Ultrason..

[7] Baldacci A., Haralabus G. Signal processing for an active sonar system suitable for advanced sensor technology applications and environmental adaptation schemes. Proceedings of the IEEE 2006 14th European Signal Processing Conference.

[8] Akyildiz I.F., Pompili D., Melodia T. (2005). Underwater acoustic sensor networks: Research challenges. Ad Hoc Netw..

[9] Ali A.M., Yao K., Collier T.C., Taylor C.E., Blumstein D.T., Girod L. (2009). An empirical study of collaborative acoustic source localization. J. Signal Process. Syst..

[10] Yu K., Zhang Y.D., Bao M., Hu Y.H. (2016). DOA Estimation From One-Bit Compressed Array Data via Joint Sparse Representation. IEEE Signal Process. Lett..

[11] Baccour N., Koubâa A., Mottola L., Iga M.A., Youssef H., Boano C.A., Alves M. (2012). Radio link quality estimation in wireless sensor networks: A survey. Acm Trans. Sens. Netw..

[12] Donoho D.L. (2006). Compressed sensing. IEEE Trans. Inf. Theory.

[13] Candes E.J., Romberg J.K., Tao T. (2005). Stable signal recovery from incomplete and inaccurate measurements. Commun. Pure Appl. Math..

[14] Candes E. (2006). Robust uncertainity principles: Exact signal reconstruction from highly incomplete frequency information. IEEE Trans. Inf. Theory.

[15] Candes E.J., Wakin M.B. (2008). An Introduction To Compressive Sampling. IEEE Signal Process. Mag..

[16] Kong L., Xia M., Liu X.Y., Wu M.Y., Liu X. Data loss and reconstruction in sensor networks. Proceedings of the IEEE INFOCOM.

[17] Bao Y., Li H., Sun X., Yu Y., Ou J. (2012). Compressive sampling-based data loss recovery for wireless sensor networks used in civil structural health monitoring. Struct. Health Monit..

[18] Charbiwala Z., Chakraborty S., Zahedi S., Kim Y., Srivastava M., He T., Bisdikian C. Compressive Oversampling for Robust Data Transmission in Sensor Networks. Proceedings of the IEEE INFOCOM.

[19] Wu L., Yu K., Cao D., Hu Y., Wang Z. (2015). Efficient Sparse Signal Transmission over a Lossy Link Using Compressive Sensing. Sensors.

[20] Donoho D.L., Elad M. (2003). Optimally sparse representation in general (nonorthogonal) dictionaries via *l*_1_ minimization. Proc. Natl. Acad. Sci. USA.

[21] Liu Z.M., Huang Z.T., Zhou Y.Y. (2011). Direction-of-Arrival Estimation of Wideband Signals via Covariance Matrix Sparse Representation. IEEE Trans. Signal Process..

[22] Luo J.A., Zhang X.P., Wang Z. A new sub-band information fusion method for wideband DOA estimation using sparse signal representation. Proceedings of the International Conference on Acoustics, Speech, and Signal processing (ICASSP 2013).

[23] Shen Q., Liu W., Cui W., Wu S., Zhang Y.D., Amin M.G. (2015). Low-Complexity Direction-of-Arrival Estimation Based on Wideband Co-Prime Arrays. IEEE/ACM Trans. Audio Speech Lang. Process..

[24] Zamalloa M.Z., Krishnamachari B. (2007). An analysis of unreliability and asymmetry in low-power wireless links. Acm Trans. Sens. Netw. Tosn Homepage.

[25] Lettieri P., Srivastava M.B. Adaptive frame length control for improving wireless link throughput, range, and energy efficiency. Proceedings of the IEEE INFOCOM.

[26] Lv X., Bi G., Wan C. (2011). The Group Lasso for Stable Recovery of Block-Sparse Signal Representations. IEEE Trans. Signal Process..

[27] SeDuMi. http://sedumi.ie.lehigh.edu/.

[28] Carlin M., Rocca P., Oliveri G., Viani F., Massa A. (2013). Directions-of-arrival estimation through Bayesian compressive sensing strategies. IEEE Trans. Antennas Propag..

